# “It’s alright to ask for help”: findings from a qualitative study exploring the information and support needs of family carers at the end of life

**DOI:** 10.1186/1472-684X-13-22

**Published:** 2014-04-17

**Authors:** Emily Harrop, Anthony Byrne, Annmarie Nelson

**Affiliations:** 1 Marie Curie Palliative Care Research Centre, Institute for Translation, Innovation, Methodology and Engagement, Cardiff University School of Medicine, Neuadd Meirionnydd, Heath Park, CF14 4YS Cardiff, UK

**Keywords:** Family caregiver, Education, Palliative care, Qualitative research

## Abstract

**Background:**

Family carers play an essential role in providing end-of-life care to their relatives but have been found to experience uncertainty and a lack of confidence in fulfilling their caregiving roles, prompting recent calls for educational or information based resources to be developed for carers.

**Methods:**

We carried out four focus groups with Clinical Nurse Specialists, healthcare assistants, former and current carers at a hospice in the UK, to explore the information and support needs of family carers.

**Results:**

Our findings support previous research by highlighting a number of care situations where carers experience uncertainty and could seemingly benefit from greater information or instruction. Three main themes were identified which reflected carer experiences and needs in relation to potential information giving or educational interventions. These have been described as the knowledge and competence of carers; the preparedness of carers and low levels of carer identification with, and confidence in their roles as ‘carers’, which influences help seeking behaviours; and in turn how potential supportive interventions might be received by carers.

**Conclusions:**

Family carers experience multiple needs for information and education, but meeting these needs remains a challenge. Our results suggest three domains which could underpin this type of intervention: developing knowledge and competence; facilitating preparedness; supporting role recognition and confidence building. We recommend an integrated information giving approach which addresses these domains by combining a resource pack for carers with a more explicit acknowledging role for health professionals. Together these could provide key information and also build confidence amongst family carers to ask for further support and advice as needed.

## Background

Family carers play an increasingly critical role in providing end-of-life care to their relatives or friends in the home setting. Ninety per cent of patients spend the majority of their last year away from hospital [1] and 55% of care needed by patients is provided by informal carers [2]. However, although there has been considerable strategic investment in supporting patient choice in terms of preferred place of care when dying, the majority of patients do not die at home. Much of the focus has been on increased professional support [3], whilst the needs of carers have been too often neglected [3-7]. The physical and psychological care needs of patients approaching the end-of-life are considerable and require specific knowledge, skills and intensive time commitment amongst those who care for them. Family caregivers commonly undertake complex care tasks including symptom assessment and management, physical comfort and medication administration [1,2]. Yet, whereas health professionals receive extensive guidance on managing such needs, when hospice patients are discharged back home families often express concerns as they themselves receive no formal training or guidance on the practicalities of physical care, instead adopting a ‘trial and error approach’ to palliative care [1].

Such experiences create barriers in terms of families’ self-perception of the care they can provide, and can precipitate re-admission to hospital and affect adherence to a patient’s choice of place of death [8]. This is not only problematic for the patient but also impacts upon carer wellbeing. A variety of psychological and physical health problems have been identified for carers some of which are associated with carers’ perception of competence and the quality of care that they provide [6,7]. Carers with better experiences of end-of-life care, including higher levels of mastery and more positive perceptions of care, have been found to be substantially less depressed over time and have better bereavement outcomes [9,10]. Drawing on a transactional model of coping, Hudson [11], similarly explains how the more capable the caregiver, or the higher the level of resources available to manage an event, the more likely are positive adaptive behaviours by the caregiver. Transactional models define coping as continually changing behavioural and cognitive efforts to manage external and/ or internal demands that are appraised as exceeding the individual’s resources [12]. Coping resources proposed by Hudson for family caregivers include feelings of preparedness, competence, having adequate information and focusing on positive aspects of the role [11]. A subsequent group education program developed and evaluated by Hudson et al. [7,13], which purposively targeted these resources, was found to be effective in preparing family carers for the role of supporting a family member to die at home.

A number of knowledge gaps have been identified in the literature. The recent systematic review by Bee et al. [1], reported a lack of knowledge about basic nursing and recommends providing training and education for the practical needs of carers. More specifically, this and other studies identified support needs in relation to practical information on nursing skills (for example hygiene assistance, using medical aids/equipment, symptom management, patient comfort and managing dietary needs), as well as access to specialist services, social support systems and services to support caregiver health, and information on the dying process and how to manage a home death [1,2,14-16]. A lack of knowledge and unrealistic expectations of the services provided by district and specialist community nurses has also been identified as a factor in carer breakdown following discharge [8].

However, research has also identified potential barriers to engaging carers in educational interventions and supportive services generally. A review of the qualitative literature by Funk et al. described how many carers express reluctance with or have difficulties acknowledging and disclosing their own needs to formal providers and asking for help [17]. In their interview study with family carers in the UK, Harding and Higginson [4], found that caregivers were ambivalent about accessing support for themselves. Carers found having nurses in the home ‘abnormal’, they felt uncomfortable leaving the patient despite expressed wishes to take a break and they lacked a sense of identification as credible service recipients. Carers reported feeling invisible or just a part of the patient, and experienced a loss of identity when caring at home. Funk et al. [17], linked this phenomenon to concerns about the legitimacy of needs, not wanting to bother formal providers, negative perceptions about and previous experiences with formal services, and a desire to keep the focus of care on the patient. A qualitative study of pain management processes of caregivers also highlights the importance of relationships with health care providers to these processes, describing how poor relationships result in feelings of isolation, frustration, helplessness, a reliance on intuition and a reluctance to ask for clarification or more information. These authors recommend an ongoing, personalised approach to carer education on pain management [16]. Hudson et al. [13], similarly note how caregivers need to be cared for in different ways, individually tailored to their specific requirements, and that whilst most are open to help and guidance, they often won't ask for it without being prompted.

This paper discusses the findings of a multiple focus group study with nurses, health care assistants and former and current family carers. The study was designed to explore the information and support needs of family carers, with a view to informing potential future interventions. This paper identifies and discusses three key domains which could be targeted to facilitate carer adaptation to their caregiving roles and finishes by making practical suggestions for what one such intervention might look like.

## Methods

The aim of the study was to explore the information and support needs of family carers, as perceived by carers and nursing staff. A qualitative method was thus selected to enable in depth exploration of experiences and perspectives, whilst focus groups were chosen as the method for data collection to facilitate group discussion and debate [18]. A critical realist approach based on in depth thematic analysis of the data was used to explore the lived experiences, meanings and the reality of family carers and nursing staff, whilst at the same time paying consideration to how participants make meaning of their experience and in turn the influence of social context on such meanings [19-21].

### The focus groups

Participants were recruited from a hospice in the UK, following ethical approval by Cardiff University School of Medicine Ethics Committee. The hospice is charity funded and provides in-patient, out-patient and home based services. The day centre manager sent out letters of invitation and participant information sheets to current carers who had been attending the day centre with their relatives and to the former carers who had attended bereavement counselling sessions at the hospice. Interested participants then contacted the team directly and suitable dates for the groups were identified. Nursing staff were approached and given participant information sheets by their managers. Written informed consent was obtained at the start of each group following further explanation of the study by the researchers and the opportunity given for participants to ask questions. Four focus groups were carried out at the hospice in September 2011. The first of the groups was with senior healthcare assistants (HCA) (n = 5), the second group was with former carers (FC) (n = 4), the third group with current carers (CC) (n = 3) and the final group was with community-based Clinical Nurse Specialists (CNS) (n = 4).

The focus groups were facilitated by two female researchers, one of whom had worked for many years as a health care assistant at the hospice prior to her current academic role. The facilitators were guided by a discussion schedule which identified set topics, questions and prompts, but allowed the discussion to develop as naturally as possible by encouraging interaction between participants. As such, the facilitator would intervene to seek the views of quieter participants, to steer the discussion along emergent lines of enquiry, and to move onto new topics when necessary. The focus groups explored:

•Views and experiences of CNS/healthcare assistants on the care provided to patients by friends and relatives at home;

•Views and experiences of carers on caring for relatives/friends in the home setting at the end of life;

•Views on the information and support needs of carers and how these might feasibly be addressed via interventions.

### The participants

The two professional groups were carried out with local teams of senior healthcare assistants and CNS following their weekly team meetings. The CNS nurses are qualified nurses who are registered to practice with the Nursing and Midwifery Council (NMC); assess, plan, deliver and evaluate all nursing care; give and can advise on prescribed medication including injections and drugs given using a syringe driver. The Senior Healthcare Assistants (SHCAs) have; training in palliative care; give care as identified in the District Nurse’s care plan; assist with personal needs such as washing, dressing and mobility; can assist with giving some routine medicines [22]. All participants were female and had a range of experience working in palliative care, from four months to seven years in the senior healthcare assistants group, and 9 months to 10 years in the CNS group (Table 1).

**Table 1:** Participants: professionals

**Participant group**	**Gender**	**Time in job (years)**	**Time working in patients’ homes (years)**	**Time in palliative care (years)**	**Professional qualifications**
Senior healthcare assistants (n = 5)	Female (n = 5)	Range = 0.25-7 years	Range = 0.25-7 years	Range = 0.3-y years	NVQ Level 3 (n = 5)
		Median = 0.3 years	Median = 0.3 years	Median = 3.5 years	
Community palliative care clinical nurse specialists (n = 4)	Female (n = 4)	Range = 0.75-5 years	Range = 0.75-27 years	Range = 0.75-10 years	BN hons (n = 1)
					MNurse sci (n = 1)
		Median = 1.75 years	Median = 8.25 years	Median = 1.75 years	Diploma in nursing (n = 2)
					BSc community health studies (n = 2)

The former carers were female widows who had been caring for their husbands (aged 56, 60, 65 and 70), and who had become good friends during bereavement counselling sessions run at the hospice over a two month period. Two of the current carers were housewives caring for their husbands and one was a daughter caring for her father (aged 39, 50, 71). This group of participants were known to the hospice because they attended the day centre with their relatives, but they were unknown to one another. With the exception of one carer who was caring for her husband with Idiopathic Pulmonary Fibrosis all other carers/former carers were caring for patients with a variety of cancers, which included bowel and liver cancer, oesophageal cancer, prostate and bowel cancer and leukaemia. The length of time caring ranged from one to seven years amongst former carers, and one year to two and a half years amongst current carers (Table 2).

**Table 2:** Participants: carers

**Participant group**	**Gender**	**Age**	**Occupation**	**Person cared for**	**Number of years caring**
Former carers (n = 4)	Female (n = 4)	Range = 56–70 years	Housewife (n = 2)	Husband (n = 4)	Range = 1–6.5 years
		Median = 62.5 years	Retired (n = 2)		Median = 3.5 years
Current carers (n = 3)	Female (n = 4)	Range = 39–71 years	Housewife (n = 3)	Husband (n = 2)	Range = 1–3 years
		Median = 50 years		Father (n = 1)	Median = 3 years

### Analysis and interpretation

The discussions were digitally recorded, transcribed verbatim and anonymised to ensure confidentiality. The transcripts were uploaded to the NVivo 8 qualitative software programme for data management and coding. The data were organised using established techniques of coding and to identify the main themes to emerge within and across groups comparison [19,23]. This was an iterative process, moving between the data and the analytical concepts to develop codes and concepts grounded in the data. Attention was also paid to group dynamics and sequences of talk to try identify areas of consensus and disagreement within the groups. Two of the transcripts (one nurse group, one carer group) were double coded to check for consistency. Inconsistencies were resolved by discussion between the two researchers. A coding framework for emergent themes was developed and validated by the research team following these discussions and was applied to the remaining two transcripts by the lead researcher with new codes being added as needed.

## Results

In this section we describe three main themes to emerge from the study, which reflected carer experiences and needs in relation to potential information giving or educational interventions. These have been described as the knowledge and competence of carers; the preparedness of carers; carer identification with, and confidence in their roles as ‘carers’, with implications for their help seeking behaviours and potential supportive interventions.

### Knowledge and competence

Feeling competent in one’s caring skills was identified as important to carer coping and stress. Given the heterogeneity of carer circumstances it is of no surprise that their experiences of care varied. However, both carers and health professionals reflected on what they felt to be limited knowledge and a lack of confidence, which could sometimes act as a barrier to the amount and type of care provided by family members.

*Well I think they all want to participate in some sort of personal care but they’re very frightened that they might do something wrong that they might hurt somebody* (HCA 4)

There were several problematic domains of care identified which included physical care, feeding and administering medication. Examples were given by nursing staff and carers of difficulties working hospital beds and of moving relatives out of chairs, after falls or to change pads.

*It’s the unpredictable things isn’t it… how do you get someone off the floor because people fall unpredictably, someone’s been incontinent how do you, how can you manage to change a pad. (…) People don’t know how to log roll… And um if the patient is still continent and needs to get to the bathroom but it’s not very easy to get out of a chair, how can you learn to stand somebody properly to get them out the chair.* (CNS 3)

*When I’ve got so many joints playing up all at once, um uh, I just say to my daughter, well you know, ‘can you try and help?’ You know, but if she’s not there I’ve just got to struggle the best I can you know even if it’s just grabbing hold of his clothing and yanking him out, many a time he’s nearly fallen on top of me. It can be a bit fiddly sometimes can’t it?* (CC2)

This and the following quote from the same carer also highlight what has been described as ‘trial and error’ approaches to caring.

*I just battle on, I think, yeah….No one showed me how to do anything…I’ve only learnt how to lift my Dad out of the chair because I used to work in a nursing home…*(CC2)

Issues were also identified with regards to feeding patients, with carers and the CNS group highlighting the role of supplement drinks in patients’ diets and the challenge of finding alternative food sources to try to broaden supplement dependent diets.

*You know we have had the dietician has come and um, I liquidise as much as I can, I make a lot of soups um and he takes the Ensure drinks. You know, he takes those um but he’s lost in a year, he’s lost over 3 stone, in fact in the last, what was it, in the last 6 weeks he lost 10 kilos* (CC3)

*What we tend to do you know with patients even a source of home food is a lot better than nothing at all but it’s still very difficult to get that information over to patients.... A lot of people they are quite kind of supplement trigger happy in the hospital…I always comparison everything and I say ‘well you know if you can’t manage your Ensure but you can manage 2 pieces of toast with butter on you’ve got the same calorie content then’* (CNS 3)

Understandably one of the most acute areas of concern for carers was with administering medication. Here examples of uncertainty and lack of confidence clearly contributed to and exacerbated reported feelings of stress, worry and anxiety.

*Yeah ‘cause when I sit down doing the medication, I will go and sit in the garden, anywhere I can get away from the television, my Dad talking, my daughter talking, it’s all at once and all that noise and I just feel like shouting ‘everyone shut up.’ Because it is so hard to concentrate, I’m petrified that I’m going to put the wrong pill in the wrong place* (CC2)

Former carers also reflected on how they would have benefited from more information on medication.

*The fact that if the pain is still there and somebody is asking you for more whom you love, you worry so then you, I needed to know more about the level of that because you are worrying that this person is not going to wake up* (FC4)

However, they also described a sense of accomplishment from eventually getting to grips with these more complex areas of care, again suggesting the psychological importance of becoming competent in care tasks.

*The carers, the amount the carers can do they might not feel they can to start with but I mean we’ve proved it here, with wives, with um epidurals and topping up. People are, OK if they don’t want to that’s another thing because some people have a fear that they are going to cause even more harm or more hurt but there are, you need to give carers I think that responsibility.* (FC4)

However, whilst the CNS participants recognized the difficulties experienced by carers when it comes to helping with symptom management and medication, they also considered that advice and education in these more complex areas could not be delivered in a universal format but would need to be tailored and responsive to individual families.

*Because we know the patients that we look after and the level of understanding that people have and there are some people who are incredibly (…) sensible (…) they understand medication very well and for other people just coping with the patient is more than enough for them to manage so I think you have to go on a case by case when you are looking at like (…) medication…* (CNS 3)

It also became apparent that a couple of carer participants were either unaware of, or not accessing, the pharmacy pre-dispensing services available, suggesting that information and advice on such services could be more systematically given out to families to help them manage this complex area of care.

### Preparedness

Feeling prepared for future events, in terms of what to expect and which services to access was also a key concern identified by and for family carers. Former carers described the value of some of the information they did eventually receive, from a reassuring as well as a practical point of view, in this case knowing ‘who does what’.

*So that, that’s one thing that could be (useful) and I think not just the carers…Well before you are in that stage where you need these people, because I didn’t need the dist’ (district nurse), I didn’t need the community nurses for a long time. Knew of them but it was really the last few weeks of his life. And uh I had to find all these things out so it would have been really helpful to have this information earlier* (FC4)

Knowledge of available services and how to access them was similarly discussed in the nursing groups. The CNS group described how a lot of families face the daunting task of having ‘to find the packages of care themselves’, whilst the healthcare assistants felt that families were sometimes unaware of their ‘sitting’ service and how to make best use of it. Discussions with current and former carers in particular highlighted the importance of accessible information on who to contact in emergency situations.

*This is what I’ve had recently, a couple of times, we sit and think, do we phone the emergency services? Do we phone here? Do we phone (names local) hospital? What do we do? So in the end I phoned the emergency service who came out and said, this was the time he had a blockage, and she said ‘well it’s Sunday now nothing will be done, leave it until tomorrow and see what happens’* (CC3)

The discussion with former carers also provided extensive descriptions and examples of how stressful experiences and perceived mistakes at the time of death, often relating to service access, caused enduring feelings of regret and blame, well into their period of bereavement.

*I just wish I hadn’t said anything and just let him have his injection and not call that GP out (on the weekend) because then perhaps on the Monday he could have (…) come here (local hospice) …. Once he got into that (…) thing of going into that hospital they needed to go through a whole process of things before they would let him (out)…. I mean at the time I thought it was all for the best but on reflection I think if only it hadn’t been a Sunday, if only I hadn’t said that* (FC4)

Carers also described anxiety over death and the dying process and the unknowns inherent in this, suggesting the importance of having good information and knowledge of illness progression.

*So my Mum had a massive stroke and she died in the same day, and that was a massive shock but it’s worse (.) looking after my Dad knowing that he is dying, every day I wake up and rush into his bedroom to check that he is still breathing* (CC2)

Others in the group also expressed concerns with finding out ‘how things are going to progress,’ although former carers agreed that they would not have liked extensive information on the dying process itself as this would have been frightening for them. Both groups of nurses were also wary of providing too much information on the dying stages as they felt that this would need to be assessed and tailored on a case by case basis.

Participants in all groups were of the view that information on financial and benefits advice, for example pensions and entitlements, help towards funeral costs and the numbers to ring to get this information, would also be useful to carers and should be straightforward to deliver.

### Identity and confidence in ‘carer’ roles

Whilst the group discussions shed some light on knowledge gaps and areas of uncertainty, there was also interesting discussion around carer self-identification and awareness of their needs, with implications for how they access support and advice. Former carers reflected upon how they experienced a ‘blinkered’ inability to recognise and respond to their own needs due to their preoccupation with the care needs of the patient and the ‘autopilot’ approach this engenders. One former carer described how “you are almost blinkered, everything is concentrated on that person you are caring for, your everyday just fades into the background.” (FC4) She went on to explain:

*Well you do get exhausted, absolutely exhausted and because you have got a routine through the day anyway so to not be able to sleep through the night because you know you are going to have the nurses coming in so you are up quickly getting ready yourself …I’m not quick in the morning but I was stuck on autopilot… and everyone thought I was managing well, but I wasn’t.* (FC4)

Following this, the former carers also suggested one of the most significant hurdles for carers to overcome is recognising that there are services available that can help them, which they could and should be accessing as credible and legitimate service users. One noted “It’s a fact of get through to somebody it’s alright to ask for help, really.” (FC1)

For one former carer one of the most important steps in her relationship with the local CNS was simply that “she gave me the confidence to ring her.” Whilst another expressed regrets that they hadn’t accessed as much support as was available, explaining “But um I know the help was there but I hadn’t quite got to it…I suppose because I thought I was coping. But I was getting very, very tired.” (FC4) Another of the former carers also highlighted the significance of her friend’s cancer experience for directing her towards the services of the hospice noting how her initial approach to the hospice “was only because of my friend you see” (FC3), thus again indicating the importance of normative factors.

Current carers also struggled to directly identify needs they might have as carers and the two wife carers did not have expectations of support for themselves. The views and experiences of the (only non-wife) daughter participant were an exception here. This was the only participant to self-describe as a ‘carer’ and to have expectations of support for herself from local services, as she described a desperate need for respite and clearly felt let down by health and social services, Both groups of carers were asked their views on a hypothetical self-referral to the palliative care service, and whereas the former carers with the benefit of hindsight perceived this as a useful idea, the current carers agreed that they would ‘never have thought’ to access the palliative care service directly themselves. In other words it is not just a lack of specific knowledge that can undermine carer perceptions of competence, but also a more general lack of confidence in their role as ‘carers’ and related to this, limited recognition of their own needs and legitimacy as service users.

The importance of more general confidence building was also discussed in the nursing groups, in particular amongst the healthcare assistants who focused more on the teaching of practical skills and the day to day experiences of carers at home, than the discussion in the CNS group which concentrated instead on which knowledge areas should or should not be included in any resource pack. These HCA participants were sceptical of any formal training package for carers and instead emphasised the need for informal, interpersonal approaches to accompany any information-based resource. They saw an opportunity for individual informal demonstrations by nursing staff either prior to discharge or during home visits, which could help build carers confidence in different care tasks whilst also providing an opportunity for learning in a less intimidating manner.

*Whether if we went up there to show them for five minutes… I mean it’s the minimal things, just a little bit of massaging of some cream or something you know, they’re scared to touch their legs in case they bruise them or something and then things like that are so comforting I think they just want somebody to show them isn’t it, to give them the confidence.* (HCA 3)

The need for informal carer-led information giving was similarly stressed in home settings too, to avoid intimidating or handing over too much responsibility to the carer.

*In the information pack saying you know, you have a senior healthcare assistant coming around, by all means ask them to show you how to do this or do you want any information about hands on care then that’s something we can help them with.* (HCA 3)

In other words it was felt that an information pack could play a role not just in conveying practical information, but also by encouraging and legitimating help seeking behaviour. This was similarly suggested in the discussion around providing ‘respite tips’ for carers. On the one hand such tips would act as practical suggestions for making the most of the ‘sitter’, but they would also help legitimate the very idea of ‘taking a break’ for the carer.

*It’s like they are waiting for permission or they are waiting to be told “yes it’s okay for you to leave, leave the house and wander off and have some me time.” It’s almost, they think that if they go off they feel guilty and they think “what must they think of me getting out of the house as soon as they arrive”.* (HCA 3)

In short, the consensus in both nurses groups seemed to favour an information giving approach which is flexible and sufficiently responsive to individual carer needs and preferences. This combination of interpersonal interaction with information resources was also suggested by the former carers, again reiterating the importance of the face to face and less formal setting.

*I think towards the end if you can, if somebody can sit down (.) you know with the carer and say when your husband dies how, how in a general way are you going to be financially because his pension or his wage will finish, are you going to be alright? Or is there anything I can talk through with you or guide you, something like that.* (FC)

Further, concerns expressed by all carers over ‘finding the time’ and difficulty leaving the patient also suggests the value of less formal and more flexible approaches to carer education interventions.

To summarise, carers and nursing staff reported uncertainty and information needs in areas such as physical care, patient comfort, hygiene assistance, equipment, symptom management and managing diet, factors we have interpreted as knowledge and competence factors. Information needs were also identified in relation to feeling prepared, including knowledge of what to expect and which services to access in given situations. However, several of the topics of concern were considered too complex and family and patient situations too variable for universal approaches. These included medication, symptom management and information on illness progression and the dying process; it was felt that health professionals would need to tailor their advice in these areas on a family by family basis. The data also revealed low levels of self-perceived need or role awareness and general confidence in this role, which further suggests the appropriateness of informal, interpersonal and personalised approaches to carer education, as also recommended by our nurse and former carer participants.

## Discussion

The focus of this research study was to explore carer support needs, and in particular the possibilities of an educational package which might address some of the knowledge gaps and training needs of carers, as identified in the literature. This research supports and adds to this literature by highlighting care tasks and situations where carers experience uncertainty and could seemingly benefit from greater information or instruction. We believe the new and most important contribution of this research, however, is in our findings of low carer confidence and self-recognition and in the practical considerations of nurses and carers of how to build confidence and support help seeking behaviour in an educational/information giving context. It was this combination of experiential and practical, problem focused discussion which took place in the focus groups which enabled us to make suggestions for a new interventional approach. In the discussion which follows we build upon our results and relevant literature to consider three interventional domains: developing knowledge and competence, facilitating preparedness and supporting role recognition and building confidence. We finish this discussion by making practical suggestions for what such an intervention might look like.

This research supports and builds upon the existing literature in several ways. As in previous research, carers and nursing staff reported uncertainty and information needs in areas such as physical care, patient comfort, hygiene assistance, equipment, symptom management and managing diet [1,2,5]. Following Hudson’s application of a transactional coping model and proposed coping resources in care-giving situations, these skill and knowledge areas could be conceptualised as competence factors [11]. Information needs were also identified in relation to feeling prepared, including knowledge of what to expect and which services to access in given situations [8], which could likewise be interpreted as preparedness factors [7,11]. Examples were also given to suggest potential outcomes linked to these coping resources. Carers and nursing staff gave examples of how uncertainty and low levels of confidence contribute to stress, worry and anxiety amongst carers, whilst examples of accomplishment and task mastery were reflected upon more positively by former carers. Conversely, descriptions of troubled death experiences caused enduring feelings of regret and guilt amongst some of the former carers with seemingly lasting impacts on their bereavement experiences. This fits with previous findings that some caregivers use positive interpretation as a meaning based coping resource [17], and that those with better experiences of end-of-life care, including higher levels of mastery and more positive perceptions of care, have been found to be substantially less depressed over time and have better bereavement outcomes [9,10].

A useful implication of this could therefore be to develop an educational resource for carers which covers topics such as; diet, personal hygiene, manual handling, respite opportunities, information on the roles of the different health and support services, and financial and benefits advice, as indeed has been recommended in previous research [1]. However, whilst some topics, such as those above might be possible to cover in such a format, our findings also strongly suggest the need for more personalised approaches to carer education and information giving. Firstly, several of the topics of concern were considered too complex and family and patient situations too variable for universal approaches. These included medication, symptom management and information on illness progression and the dying process. While there are indications that families would benefit from greater information on these topics it seems that health professionals would need to tailor their advice on a family by family basis. Secondly, issues around more general carer confidence and low levels of self-perceived need, role awareness and a reluctance to seek help, also previously highlighted [4,16,17], further suggests the importance of informal, interpersonal and personalised approaches to carer education. Such approaches were explicitly discussed by our nurse and to a lesser degree carer participants, and are in line with some of the recommendations made in the discussed literature for ongoing personalised education [13,16,17]. Indeed, there may be as much benefit to come from building confidence in carers to ask for assistance and support as from the specific competencies developed from effective task mastery and the instruction itself. Such approaches could therefore serve a dual role of both enabling carer knowledge and competence in specific care domains, but also facilitating carer identification with, and more general confidence in their roles as ‘carers’, and in turn their own self perceived credibility and legitimacy to ask for support and advice.

In terms of putting this approach into practice, there are some general messages for how health professionals involved in patient care at the end of life engage with and support family carers. In their routine interactions with patients and their families, health professionals could look to more explicitly encourage requests for help and advice from relatives. This might mean making extra effort to acknowledge the role played by family carers and the challenges associated with this, could include prompting for common areas of concern or providing carers with a list of topics that they can provide help and advice with.

However, these results also suggest an opportunity to develop a more structured package or intervention which combines the kind of professional approach outlined above with an information pack for carers. Such a pack could provide practical information, advice and tips for those topics where it is feasible to do this, as identified above. The more complex topics identified could also be presented, but as subjects on which carers can request further information and advice from their appropriate health professionals. By acknowledging the role of family carers and consistently encouraging carers to ask for further information or support for each of the different topic areas, such a package could help legitimate given topics as valid areas for discussion, whilst more generally also helping to give carers a sense of legitimacy as credible seekers of information, help and support. These messages in turn need to be reinforced by key health professionals who could use the package as an aid or set of prompts; as a way of explicitly acknowledging the role of the carer, to reiterate the additional support and advice which they can offer or to explicitly check if there are any areas which they could benefit from more information on.

Such a package thus shares some of the same rationale as that identified for the recently developed Carer Support Need Assessment Tool (CSNAT) [15]. Both are intended to help make explicit carers needs for themselves, and both recognise the potential value of having a structured tool which can act as an aid for health professionals to assess and meet the needs of carers [15]. As an educational package (as opposed to assessment tool), this proposed approach addresses those important domains of carer confidence and self-recognition, whilst at the same time helping carers to develop their competence and preparedness for their caregiving journeys. Whilst previous ‘one to one’ interventions for carers have been noted to be useful but expensive, and group interventions can be difficult for some carers due to the requirement to leave the patient [13,14,24], such an approach could be accommodated as part of routine care for patients and families.

Further, the relative cost of developing such a resource combined with guidance or training for associated health professionals, could in the long run represent a considerable saving when set against the high costs of hospital readmissions following the breakdown of family care, with implications also for the health, wellbeing and bereavement outcomes of family carers.

### Study limitations and implications for research

A limitation of this study was the low numbers of participants in the focus groups, in particular the carer groups. Of these it should also be noted that all but one of the carers were ‘wives’, meaning that our results predominantly represent a female spousal experience and perspective as opposed to laying claim to any kind of universal carer experience. Indeed the data suggested some strong differences between the daughter and wife experiences in terms of how they acknowledged and identified with an explicit ‘carer’ identity. For example, the daughter was the only participant to self-describe as a ‘carer’ and articulate her own needs as a carer. Further, all carers were of white British origin and it is likely that the experiences of different ethnic groups, male and filial caregivers will vary from our results. Future research should seek to explore the experiences of such groups. The fact that all participants were recruited from only one hospice, and in particular that all of the former carers were identified through a bereavement counselling group, might also have influenced the reported experiences of these groups.

Although the small numbers were disappointing, this was not unexpected given the time constraints on family carers and the sensitive nature of the topic area, whilst the numbers in our nursing groups were determined by the size of the team present at the hospice on those days. Due to the high time demands on both staff and carers we felt that a pragmatic approach to data collection with these populations was justified, although it is acknowledged that the inclusion of further sites would have helped with recruitment and strengthened our findings Nonetheless, the quality and richness of our data suggested some positives to the small group sizes. Carers were afforded greater opportunity and perhaps a more comfortable environment to tell their stories than would have been the case in larger groups, but at the same time were also encouraged to talk by their interaction with the group. It was this useful combination of experiential and practical, problem focused discussion which took place in the focus groups which enabled us to outline an interventional approach. Finally, although the individual group sizes were small we nonetheless had engaging and informative conversations with 16 people with considerable experience and knowledge of the topic area.

In terms of messages for future research, the development of this kind of package requires further exploratory research. An in depth observational study of how information and advice is currently given to families by their health professionals in different health care settings (eg at home, hospices, GP practices) would be useful to help further our understandings of the strengths, limitations and opportunities of these different interactive settings. Future research interested in developing and evaluating any such package would clearly also need to invest considerable time in a comprehensive developmental and piloting phase to investigate not only the optimal content of the resource but also the different options for implementation, as per the MRC Guidance for developing and evaluating complex interventions [25].

## Conclusion

Family carers experience multiple needs for information and education, but meeting these needs through approaches which are accessible and acceptable to carers remains a challenge. Our results suggest three domains which could underpin this type of intervention; developing knowledge and competence; facilitating preparedness and supporting role recognition and confidence building. We propose an integrated information giving approach which addresses these domains by combining a resource pack for carers with a more explicit acknowledging role for health professionals. Together these could provide key information and also build confidence amongst family carers to ask for further support and advice as needed.

## Abbreviations

CC: Current carers; CNS: Clinical nurse specialists; CSNAT: Carer support need assessment tool; FC: Formal carer; GP: General practitioner; HCA: Health care assistant; NMC: Nursing and midwifery council; SHCA: Senior health care assistants.

## Competing interests

The author(s) declare that they have no competing interests.

## Authors’ contributions

EH facilitated the focus groups, analysed the data and drafted the paper. AN facilitated the focus groups and helped to draft the paper. AB conceived of the study, participated in its design and coordination and helped to draft the manuscript. All authors read and approved the final manuscript.

## Pre-publication history

The pre-publication history for this paper can be accessed here:

http://www.biomedcentral.com/1472-684X/13/22/prepub
